# Structural changes in femoral bone tissue of rats after subchronic peroral exposure to selenium

**DOI:** 10.1186/1751-0147-55-8

**Published:** 2013-02-01

**Authors:** Monika Martiniaková, Ivana Boboňová, Radoslav Omelka, Birgit Grosskopf, Robert Stawarz, Róbert Toman

**Affiliations:** 1 Department of Zoology and Anthropology, Constantine the Philosopher University, Nitra, 949 74, Slovakia; 2 Department of Botany and Genetics, Constantine the Philosopher University, Nitra, 949 74, Slovakia; 3 Institute of Zoology and Anthropology, Georg-August University, Göttingen, 37 073, Germany; 4 Institute of Biology, Krakow Pedagogical University, Krakow, 31 054, Poland; 5 Department of Veterinary Sciences, Slovak University of Agriculture, Nitra, 949 76, Slovakia

## Abstract

**Background:**

The role of selenium (Se) on bone microarchitecture is still poorly understood. The present study aims to investigate the macroscopic and microscopic structures of femoral bone tissue in adult male rats after subchronic peroral administration of Se.

**Methods:**

Twenty one-month-old male Wistar rats were randomly divided into two experimental groups. In the first group (Se group) young males were exposed to 5 mg Na_2_SeO_3_/L in drinking water, for 90 days. Ten one-month-old males without Se administration served as a control group. At the end of the experiment, macroscopic and microscopic structures of the femurs were analysed using analytical scales, sliding instrument, and polarized light microscopy.

**Results:**

The body weight, femoral length and cortical bone thickness were significantly decreased in Se group rats. These rats also displayed different microstructure in the middle part of the femur, both in medial and lateral views, where vascular canals expanded into the central area of the bone while, in control rats, these canals occurred only near the endosteal surfaces. Additionally, a smaller number of primary and secondary osteons was identified in Se group rats. Histomorphometric analyses revealed significant increases for area, perimeter, maximum and minimum diameters of primary osteons’ vascular canals but significant reductions for all measured variables of Haversian canals and secondary osteons.

**Conclusions:**

Se negatively affected the macroscopic and microscopic structures of femoral bone tissue in adult male rats. The results contribute to the knowledge on damaging impact of Se on bone.

## Background

Bone is a specialized connective tissue, which forms the framework of the body. Its metabolic activities may be immediate targets for xenobiotics and various physiological conditions can adversely affect bone metabolism leading to skeletal deformities and diseases. Excess or deficiency of certain essential elements may also affect bone maturation [1,2].

Selenium (Se), an essential trace element, plays an important role in mammalian biology [3]. It is known that humans and animals require Se for the normal function of a number of Se-dependent biological processes [4,5]. At low concentration (≤ 0.3 mg/L), Se exerts its various effects through its incorporation into selenoproteins. According to Ebert and Jakob [6] several selenoproteins are expressed in bone and play important roles in bone metabolism. Se deficiency may induce retardation of bone formation [7] or may be a possible risk factor for osteoporosis [6]. At higher concentrations (≥ 4 mg/L), however, Se is cytotoxic. It is widely known that high concentrations of Se trigger the opening of the mitochondrial permeability transition pore by inducing the synthesis of the superoxide anion and the oxidation of protein thiol groups [8-10]. Chung *et al.*[11] found that sodium selenite induced apoptosis in mature osteoclasts mediated by mitochondria. Excess of Se also causes the death of osteoblast-like cells [12], leads to abnormal bone and cartilage development and is reported to be teratogenic [13].

Studies of the effects of Se on bone structure contribute to the knowledge on the mechanisms of Se activity in the organism and relations between changes found in different organs. Generally, the effects of Se on bone tissue are studied mainly in relation to the deficiency of Se in the food, but it is also interesting if and how Se can impact on bone structure and function after subchronic intoxication. Administration of subchronic toxic doses allows for the high probability that the toxic effect will be observable and defined at the structural level. In our study the subchronic peroral dose was used to study toxic effects of Se on macroscopic and microscopic structures of femoral bone tissue in rats.

## Methods

### Animals

Twenty one-month-old male Wistar rats were obtained from the accredited experimental laboratory (number SK PC 50004) of the Slovak University of Agriculture in Nitra (Slovakia). These clinically healthty rats were randomly divided into two experimental groups of 10 individuals. Male rats were used, as they are less susceptible than females to Se toxicity. The rats were housed individually in plastic cages in an environment maintained at 20-24°C, 55 ± 10% humidity. They had access to water and food (feed mixture M3, Bonargo, Czech Republic) *ad libitum*. The first group (Se group, n=10 rats) was exposed daily to 5 mg Na_2_SeO_3_/L in their drinking water for a total of 90 days. The water consumption was monitored daily during the whole experiment. The selenium intake was calculated from the water consumption and expressed as total amount of consumed Se. The average amount of consumed Se was 9.7 mg per animal. The dose was high enough to reach toxicity but also safe enough to prevent animal mortality. The second group (n=10 rats), without Se exposure, served as the control group. This study was approved by the Animal Experimental Committee of the Slovak Republic.

### Procedures

At the end of the 90 days, all the rats were killed and their femurs were used for macroscopic and microscopic analyses. The left femurs were weighed on analytical scales with an accuracy of 0.0001 g and the length was measured with a sliding instrument.

For histomorphometric analysis, the right femurs were sectioned at the midshaft of the diaphysis and the segments fixed in HistoChoice fixative (Amresco, USA). The segments were then dehydrated in increasing grades (40 to 100%) of ethanol and embedded in Biodur epoxy resin (Günter von Hagens, Heidelberg, Germany) according to the method described by Martiniaková *et al.*[14]. Transverse thin sections (70–80 μm) were prepared with a sawing microtome (Leitz 1600, Leica, Wetzlar, Germany) and fixed onto glass slides by Eukitt (Merck, Darmstadt, Germany) as previously described [15]. The qualitative histological characteristics of the compact bone tissue were determined according to the internationally accepted classification systems of Enlow and Brown [16] and Ricqlés *et al.*[17], who classified bone into three main categories: primary vascular tissue, non-vascular tissue and Haversian bone tissue. Various patterns of vascularization can occur in primary vascular bone: longitudinal, radial, reticular, plexiform, laminar, lepidosteoid, acellular, fibriform and protohaversian. There are three subcategories indentified in Haversian bone tissue: irregular, endosteal and dense.

The quantitative (histomorphometric) variables in the anterior, posterior, medial and lateral views of thin section(s) were assessed using the software Motic Images Plus 2.0 ML (Motic China Group Co., Ltd.). We measured area, perimeter and the minimum and maximum diameters of the primary osteons’ vascular canals, Haversian canals and secondary osteons in all views of the thin sections in order to minimize inter-animal differences.

Diaphyseal cortical bone thickness was also measured by Motic Images Plus 2.0 ML software. Twenty random areas were selected, and average thickness was calculated for each femur.

### Statistics

Statistical analysis was performed using SPSS 8.0 software. All data were expressed as mean ± standard deviation (SD). The unpaired Student’s *t*-test was used for establishing statistical significance (*P* < 0.05) between experimental groups.

## Results

### Macroscopic differences

Body weight and femoral length were significantly decreased in the Se-exposed rats compared to the control rats. Cortical bone thickness was also significantly lower in rats from the Se group. However, femoral weight was not different between the two groups (Table 1).

**Table 1:** **Body weight, femoral weight, femoral length and cortical bone thickness in adult male rats subchronic exposed to 5 mg of Na**_**2**_**SeO**_**3**_**/L in drinking water for 90 days (Se group) and control rats**

**Group**	**N**	**Body weight (g)**	**Femoral weight (g)**	**Femoral length (cm)**	**Cortical bone thickness (mm)**
Control	10	405.0 ± 52.7	1.05 ± 0.17	3.94 ± 0.09	0.572±0.054
Se	10	352.5 ± 37.1	1.07 ± 0.11	3.74 ± 0.07	0.528±0.051
*T*-test		*P* < 0.05	NS	*P* < 0.05	*P* < 0.05

N: number of rats, NS: non-significant changes.

### Microscopic differences

**Figure 1:**
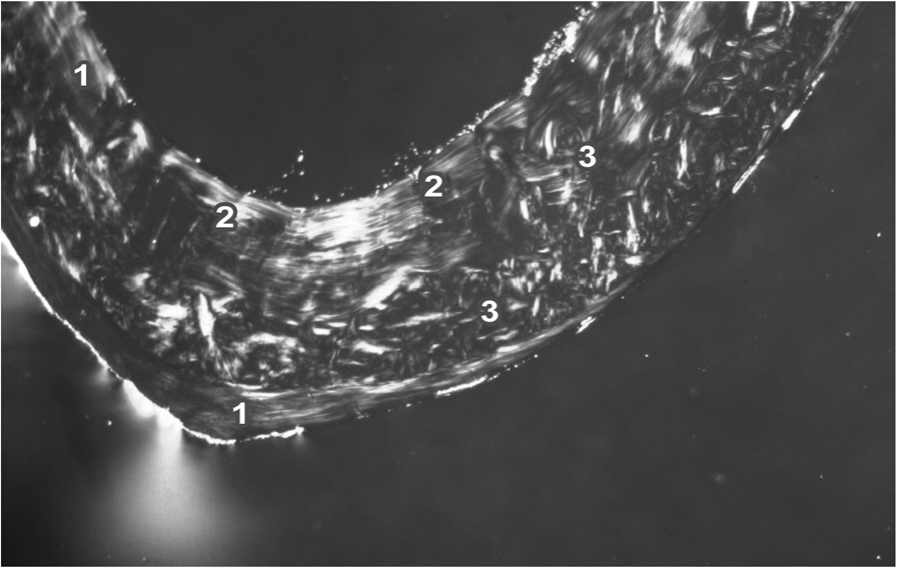
**Microscopic structure of compact bone tissue in rat from the control group (antero-lateral view).** 1 non-vascular bone tissue. 2 vascular canals radiating from marrow cavity. 3 primary and secondary osteons in middle part of compact bone.

**Figure 2:**
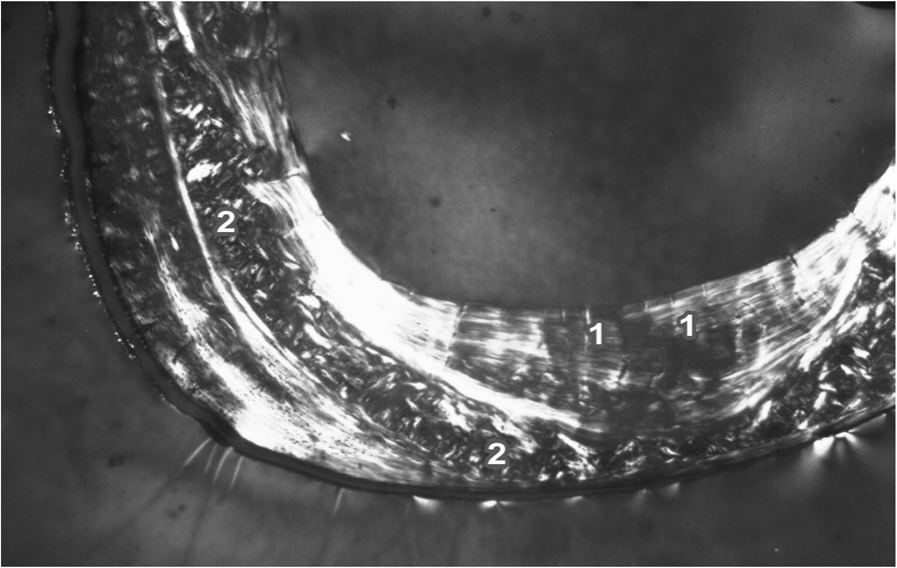
**Microscopic structure of compact bone tissue in rat from the Se group (antero-lateral view).** 1 Enormous vascular canals radiating from marrow cavity. 2 Smaller number of primary and secondary osteons in middle part of compact bone.

The endosteal border of the femurs from the control rats was formed by non-vascular bone tissue in all views of the thin sections. The bone tissue contained cellular lamellae and osteocytes. Primary or secondary osteons were absent. Areas of primary vascular radial bone tissue were also identified in anterior, posterior and lateral views. Branching or non-branching vascular canals radiating from the marrow cavity were also detected. Some primary and secondary osteons were found especially in the anterior and posterior views near the endosteal surfaces. In the middle part of the compact bone, a few primary and secondary osteons were identified. The periosteal border was again composed of non-vascular bone tissue, mainly in the anterior and posterior views (Figure 1). The Se-exposed rats displayed a similar microarchitecture to that of control rats, except for the middle part of the femur in the medial and lateral views. Vascular canals were shown to have expanded into the central area of the bone in these views. In some cases, the expansion was enormous and the canals also occurred near periosteal surfaces. A smaller number of primary and secondary osteons was also identified in the Se-treated rats (Figure 2).

For the quantitative histological characteristics, 421 vascular canals of primary osteons, 411 Haversian canals and 411 secondary osteons were measured in total. Results are summarized in Table 2. We found that all measured variables (area, perimeter, maximum and minimum diameters) of the primary osteons’ vascular canals were higher in the Se-exposed rats than in control rats (*P*<0.05). However, these rats displayed significantly decreased levels of all variables of Haversian canals and secondary osteons (*P*<0.05).

**Table 2:** **Data of the primary osteons’ vascular canals, Haversian canals and secondary osteons in adult male rats subchronic exposed to 5 mg of Na**_**2**_**SeO**_**3**_**/L in drinking water for 90 days (Se group) and control rats**

	**Group**	**N**	**Area (μm**^**2**^**)**	**Perimeter (μm)**	**Max. diameter (μm)**	**Min. diameter (μm)**
**Primary osteons’ vascular canals**	Control	218	397.3 ± 98.1	72.29 ± 8.95	12.89 ± 2.08	9.83 ± 1.58
	Se	203	459.6 ± 127.5	77.93 ± 11.11	14.08 ± 2.58	10.36 ± 1.51
	*T*-test		*P* < 0.05	*P* < 0.05	*P* < 0.05	*P* < 0.05
**Haversian canals**	Control	208	426.9 ± 119.2	74.47 ± 10.25	13.21 ± 2.16	10.24 ± 1.73
	Se	203	375.5 ± 67.4	70.29 ± 6.58	12.49 ± 1.68	9.64 ± 1.28
	*T*-test		*P* < 0.05	*P* < 0.05	*P* < 0.05	*P* < 0.05
**Secondary osteons**	Control	208	6541.0 ± 2012.6	291.79 ± 43.09	52.21 ± 8.61	39.38 ± 7.52
	Se	203	5766.7 ± 1539.2	272.16 ± 35.81	47.80 ± 7.06	37.99 ± 6.24
	*T*-test		*P* < 0.05	*P* < 0.05	*P* < 0.05	*P* < 0.05

N: number of measured structures.

## Discussion

Subchronic peroral exposure to 5 mg Na_2_SeO_3_/L in drinking water for 90 days resulted in a significant decrease in body weight and femoral length in adult male rats. Ip [18] similarly reported a reduction in growth in rats after 12 weeks of dietary exposure to 5 mg/kg Na_2_SeO_3_. Selenium is known to accumulate in the anterior part of the pituitary gland. Thorlacius-Ussing *et al.*[19] mention decreased secretion of growth hormone (GH) and somatomedin C in rats after receiving 15 mg/L Na_2_SeO_3_ in their drinking water, suggesting that growth retardation could be mediated by reduced GH and somatomedin C production. Next Gronbaek *et al.*[20] showed that a Se dose of 3.3 mg Na_2_SeO_3_/L in drinking water for 35 days induced a significant reduction in circulating insulin-like growth factor I (IGF-I). These rats also displayed a significantly shorter tibia.

The thickness of cortical bone is generally accepted as an important parameter in the evaluation of cortical bone quality and strength. We observed significantly decreased cortical bone thickness in Se-exposed rats. Studies of cortical bone thickness in Se-exposed rats has not been published previously. The value of cortical bone thickness in control group rats differed from values reported by Comelekoglu *et al.*[21] and Chovancová *et al.*[22], who analysed rats of different age.

The results of the qualitative histological analysis of the control rats corresponded to those of previous works [23-26]. We identified non-vascular and primary vascular radial tissues and irregular Haversian bone tissue. However, there was no evidence of true Haversian intracortical bone remodelling. It is generally known that aged rats and mice lack true Haversian cortical bone remodelling but not cancellous bone remodelling [25,27]. Therefore, some secondary osteons can be observed in the long bones near the endosteal border. In our study, the newly formed remodelling units within compact bone originated from the endocortical surface and extended deep into the underlying compact bone. The same findings have also been documented in 13 month-old male rats [25].

Prolonged intake of a high dose of Se induced changes in the middle part of compact bone where primary vascular radial bone tissue was present. We propose that the formation of this type of bone, in the central area of the femur, could be explained as an adaptive response to Se toxicity to protect bone tissue against cell death. It is generally known that Se at high doses induces apoptosis in mature osteoclasts [11] and death of osteoblast-like cells [12]. In a study by Turan *et al.*[28] osteocyte loss was identified because of the destruction of the bone tissue and its replacement with a large uncalcified mass of new bone matrix in rabbits fed excess Se (10 mg Na_2_SeO_3_/kg of diet for a period of 12 weeks). These authors also reported decreased biomechanical strength of the femur in the Se-exposed animals.

Data obtained from the histomorphometric analysis showed a significant increase in area, perimeter, maximum and minimum diameters of the primary osteons’ vascular canals and on the other hand a significant decrease of the Haversian canals’ variables in the Se-exposed rats. In general, the vascular system is a critical target for toxic substances and their effects on the vascular system may play an important role in mediating the pathophysiological effects of these substances in specific target organs [29]. Blood vessels readily adapt structurally in response to sustained functional changes, particularly those related to chronic pressure alterations or changes in the nutritional demands of the tissue [30,31]. Results obtained by Ruseva *et al.*[32] demonstrate that rats exposed to increased amount of dietary Se had higher glutathione peroxidase 1 (GPx-1) activity and a lower level of anti-elastin antibodies (AEABs) than those of control group, and the aortic wall histology showed degenerative changes associated with reduced thickness of the wall of the left coronary artery. Our results indicate that an excess of Se had a different impact on the primary osteons’ vascular canals and Haversian canals. The main difference between these structures is the presence of a cement line in Haversian canals (cement line delimits the canals) and its absence in primary osteons. We surmise that the cement line could be the main reason for the different results in the histomorpometry of both canals.

We found significantly lower values of all variables of secondary osteons in Se-exposed rats. According to Jowsey [33], the values of secondary osteons are higher in individuals with longer bones. Our results correspond with those found by Jowsey [33], as the femurs were longer in the control rats, which also displayed higher values for osteons. Moreover we propose that the observed differences in histomorphometry between the Se-exposed and control rats could be associated with changes in bone remodelling, which is mediated by osteoblasts and osteoclasts and subsequent calcification of bone tissue. The results of Boyar [34] showed that an excess of Se increased the amount of carbonate content in bones of Wistar rats injected intraperitoneally with 5 μmol Na_2_SeO_3_/kg for 4 weeks. The incorporation of carbonate ions into the crystal structure of hydroxyapatite (HA) results in changes in the physical and chemical properties of HA [35]. HA crystals, as a major mineral component of bones, are aligned with their long axis parallel to the collagen fibre axis [36] creating concentric lamellae of secondary osteons. Another factor that could have contributed to the minimization of secondary osteons could be Se intoxication, which induces apoptosis in mature osteoclasts [11]. Decreased osteoclast activity is associated with smaller osteon size [37]. Therefore, significantly decreased secondary osteons may be present in Se-exposed rats.

The paper is the first report of femoral bone structure in rats after subchronic peroral administration of Se. Our results contribute to the insight into the complexity of Se toxicology in rats. Moreover, since the microstructural changes in the bone typically follow biochemical action of Se in the tissue, the results of the study can highlight the physiological implications and possible mechanisms of Se effects.

Our results demonstrate the effect of Se on bone microstructure of rats; however, possible extrapolation of the results to humans may be an interesting topic for discussion. Although Se poisoning is not reported frequently in humans and human toxicity from environmental exposure to Se is rare, incidents include industrial accidents, accidental ingestions, suicides, and attempted murder [38]. In humans, the minimum lethal dose seems to be similar to that for animals. The chronic toxic dose for human beings is about 2.4 to 3 mg of Se per day [39]. Although there are some differences in bone microstructure between humans and rats we suppose that observed changes in the variables of Haversian canals and secondary osteons in our rats will correspond to those of humans. Human femur in adult individuals is namely composed of many secondary osteons, which contain Haversian canals (dense Haversian bone tissue). Additional research dealing with the influence of Se on human bone structure is required to obtain more information on comparative aspects.

## Conclusions

This study demonstrates that subchronic peroral administration of 5 mg Na_2_SeO_3_/L in drinking water affects the body weight, femoral length, cortical bone thickness and both the qualitative and quantitative microscopic structures of femoral bone tissue in adult male rats. The results can be applied in experimental studies focusing on the effects of various trace elements on bone structure.

## Competing interests

The authors declare that they have no competing interests.

## Authors’ contributions

MM was responsible for microscopical analysis of bones. IB was responsible for macroscopical analysis. RO was responsible for the statistical analysis. BG was responsible for preparation of histological sections. RS was responsible for photodocumentation of histological sections. RT was responsible for animal care and sampling of femora. All authors read and approved the final manuscript.
